# Unusual Metastasis of Gastric Signet Ring Cell Carcinoma to the Breast: A Case Report of a Young Moroccan Patient

**DOI:** 10.7759/cureus.56333

**Published:** 2024-03-17

**Authors:** Mohammed Bendimya, Mouna Kairouani, Mohammed El Magroud, Amal Bennani, Ouissam Al Jarroudi, Sami Aziz Brahmi, Said Afqir

**Affiliations:** 1 Medical Oncology, Faculty of Medicine and Pharmacy of Oujda, Oujda, MAR; 2 Medical Oncology, Mohammed VI University Hospital, Mohammed First University of Oujda, Oujda, MAR; 3 Anatomopathology, Faculty of Medicine and Pharmacy of Oujda, Oujda, MAR

**Keywords:** immunotherapy, chemotherapy, immunohistochemistry, breast cancer, signet-ring cell, breast metastasis, gastric cancer

## Abstract

Although gastric cancer is known to be an aggressive tumor that can spread throughout the body, breast metastases are uncommon. This entity is rarely reported in the literature, with an estimated incidence of 0.5 to 1.3%. We report a case of a rare association between a gastric subtype of signet ring cell carcinoma and metastasis to the breast. This uncommon situation is only documented through case reports. Most breast metastases have been detected after diagnosis of primary gastric cancer, during the first year. Several risk factors have been suggested to explain the aggressive behavior of these tumors, which correlates with very poor prognosis and short survival. We report the case of a 22-year-old female patient presenting with widespread metastatic gastric signet ring cell carcinoma with an unusual secondary site in the breast. The diagnosis was confirmed by immunohistochemistry (IHC) and radiology, and the patient was treated with palliative chemotherapy in accordance with the decision of the multidisciplinary tumor board.

## Introduction

Breast metastases from an extra-mammary primary site, such as gastric carcinoma with signet ring cells, are uncommon and have an incidence of less than 2% [1,2]. Young women are most commonly affected by this presentation [3,4], with a median age at diagnosis of 46 years, in contrast to primary breast cancer which appears at a median age of 61 years [5], they are less sensitive to chemotherapy (ChT) [6,7], and are associated with a poor prognosis and low overall survival (OS).

## Case presentation

We report the case of a young 22-year-old patient with no family history of cancer. The patient presented with postprandial vomiting four months before her diagnosis without any other signs of digestive symptoms, associated with diffuse and intermittent bilateral rib pain evolving in a context of deterioration of the general condition made up of weight loss estimated at 20 kilograms in two months.

**Figure 1:**
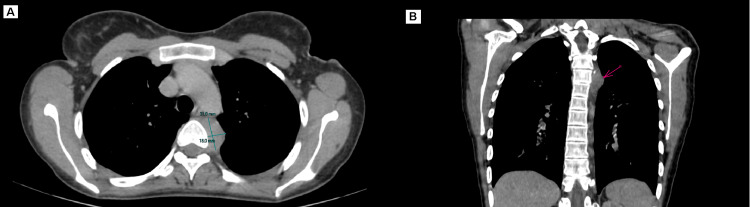
Axial (A) and Coronal (B) CT scan images (arrow) Showing a posterior mediastinal, retroesophageal and prevertebral mass at the level of D4, in close contact with the descending thoracic aorta and the vertebral body of D4.

**Figure 2:**
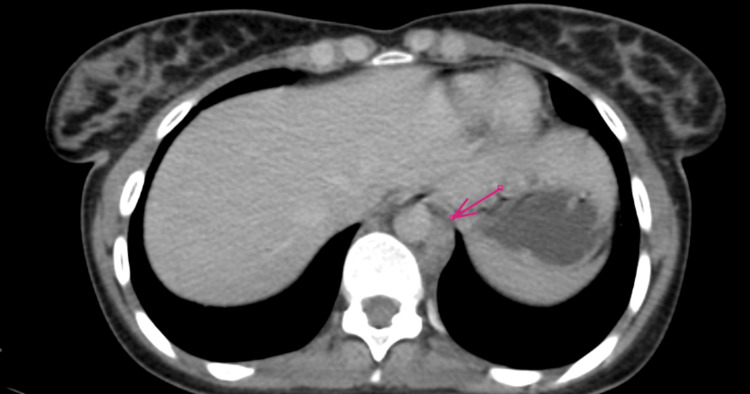
Axial CT scan images (arrow) Showing supradiaphragmatic mass adhering to the descending aorta and abdominal aorta, in close contact with the vertebral bodies of D11 and D12.

The patient initially underwent a thoraco-abdomino-pelvic computed tomography (CT) scan which revealed the presence of a posterior retro-esophageal and pre-vertebral mediastinal mass measuring 30mm x 21mm at the level of the D4 vertebral body with an endo-foraminal extension, as well as a second mass supra and infra-phrenic in close contact with the descending aorta, abdominal aorta and vertebral body D11-D12, measuring 32mm x 16mm; these two masses were associated with mesenteric and retroperitoneal lymphadenopathy, which initially suggested a hematologic lymphoma (Figures 1, 2).

A costovertebral biopsy was performed, followed by an immunohistochemical study, showing intense and diffuse positive staining for antibody (AB) anti-AE1/AE3 (+) and anti-CK7 (+) with negative staining for AB anti-CK20 (-), anti-CK 5/6 (-), anti-TTF1 (-), anti-GATA3 (-), anti-RO (-), anti-RP (-), anti-HER2 (-), and anti-calretinin (-), in favor of a carcinomatous process primarily of gastric or mammary origin.

**Figure 3:**
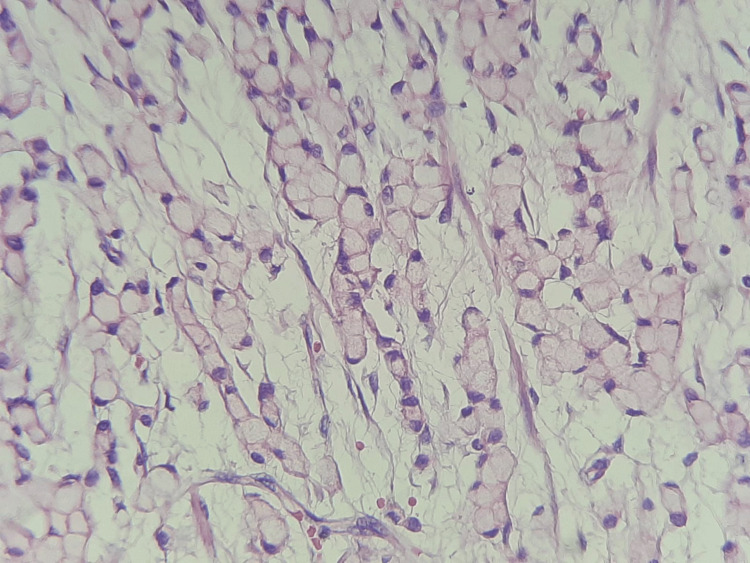
Pathological findings of gastric biopsy Microphotography showing proliferation of cells with abundant clear cytoplasm and eccentric hyperchromatic nuclei, giving a signet ring cell appearance (hematoxylin and eosin, x200).

An oeso-gastro-duodenal fibroscopy (FOGD) was subsequently performed, which revealed fundic thickening with the presence of a hypertrophic fundic fold at the antro-fundiac junction, a number of biopsies had been taken from the stomach, confirming the presence of signet ring cell gastric cancer, associated with the presence of Helicobacter pylori, and that tumor was mismatch repair proficient (Figure 3).

After a multidisciplinary tumor board, we concluded that the patient had widespread metastatic signet ring cell gastric cancer to the bone, and lymph nodes and would therefore be a candidate for palliative chemotherapy with assessment.

The patient was subsequently lost to follow-up. Three months later, the patient reported a deterioration in health with a performance status (PS) of 2, epigastric pain, vomiting, and back pain. Clinical examination revealed a painless mass in the upper outer quadrant of the left breast measuring 3 cm in diameter.

**Figure 4:**
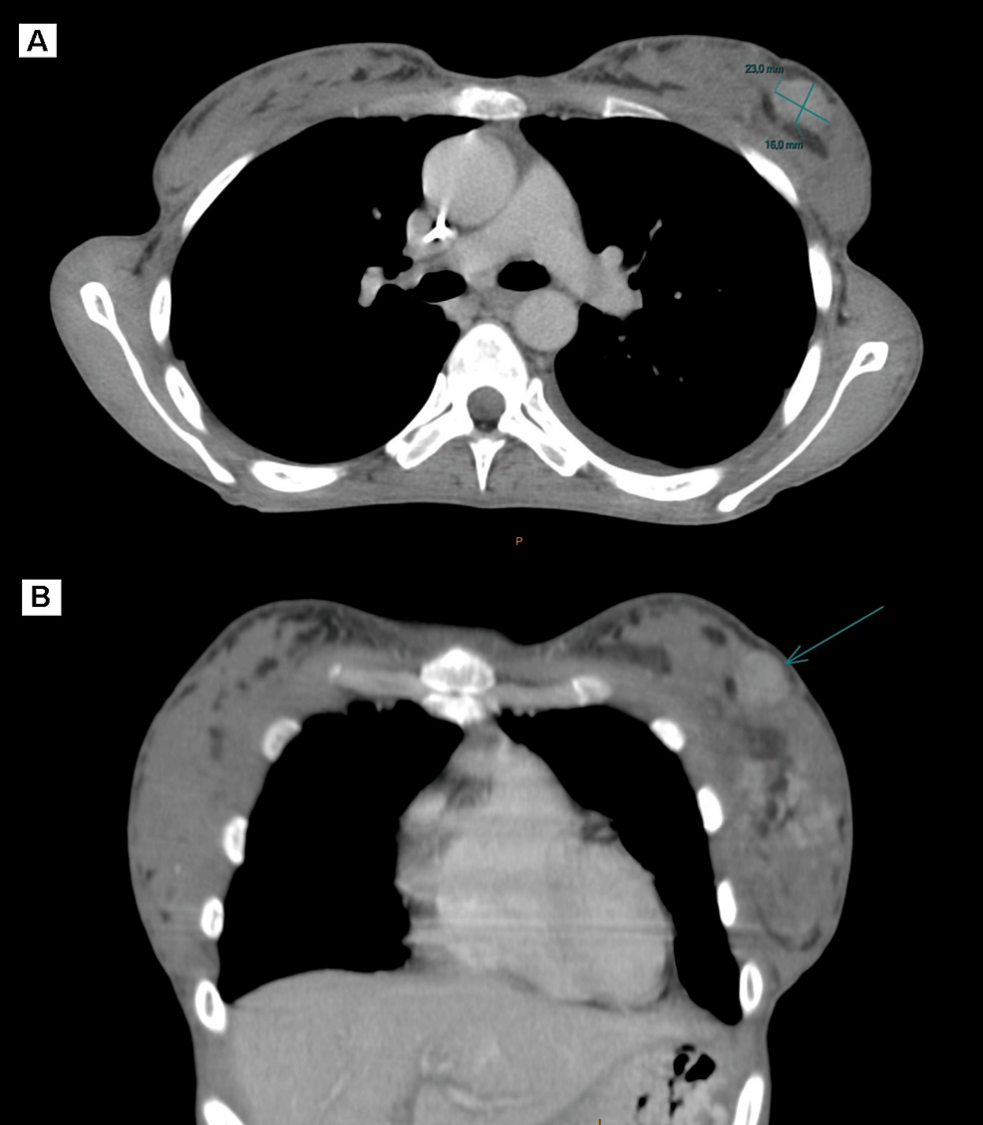
Axial (A) and Coronal (B) CT scan images (arrow) Showing a breast metastasis in the upper outer quadrant of the left breast with a diameter of 26 mm.

**Figure 5:**
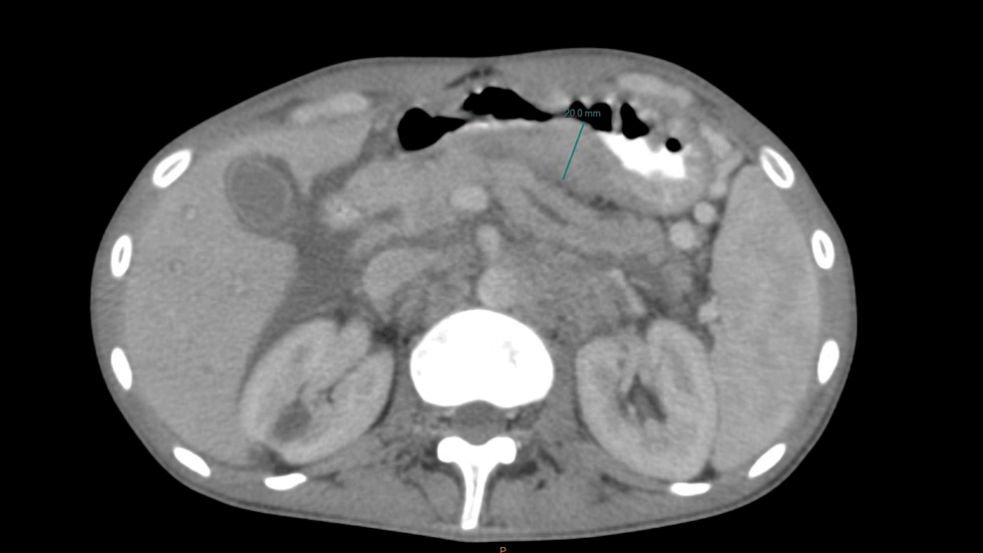
Axial CT scan images Showing the primary gastric tumor located in the posterior wall of the antrum with a thickness of 20 mm.

The new thoraco-abdomino-pelvic CT-scan showed a multifocal tumor process in the left breast, the largest of which was located in the upper outer quadrant measuring 26 mm (Figure 4). It was associated with the presence of a gastric tumor process, peritoneal carcinomatosis, multiple secondary bone lesions of the axial skeleton, and malignant lymph nodes in the supraclavicular, mediastinal, diaphragmatic, and retroperitoneal areas (Figure 5).

**Figure 6:**
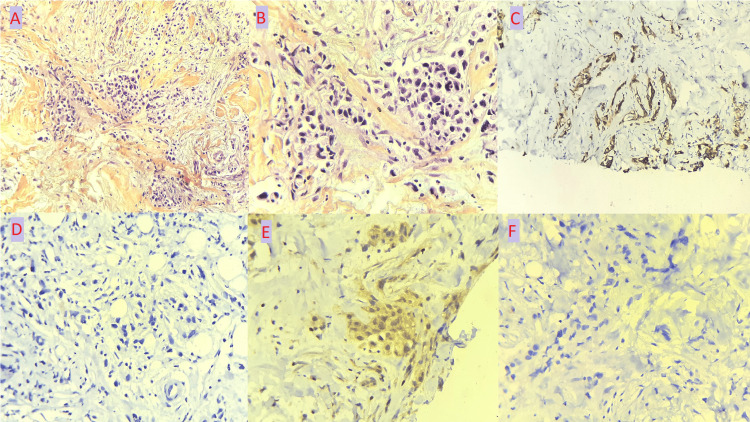
Histopathological and immunohistochemical findings of breast metastasis biopsy (A) Breast tissue, site of a carcinomatous tumor proliferation arranged in glands, nests, trabeculae and isolated cells (haematoxylin and eosin,x200), (B) Pleomorphic tumor cells with abundant and eosinophilic cytoplasm. The nucleus is large and hyperchromatic. In some places the tumor cells have a clear cytoplasm and an eccentric nucleus giving a signet-ring cell appearance (haematoxylin and eosin,x400), (C) Positive staining of tumor cells by the anti-CK7 antibody (immunohistochemical staining,x200), (D) Lack of staining of tumor cells by the anti-CK20 antibody (immunohistochemical staining,x400), (E) Cytoplasmic staining (negative) of tumor cells by the anti-GATA3 antibody (immunohistochemical staining,x400), (F) Lack of staining of tumor cells by the anti-mammaglobin antibody (immunohistochemical staining,x400).

A tru-cut biopsy of the left breast has shown a histological appearance of adenocarcinoma with an independent cellular component with immunohistochemical profile showing positive staining of tumor cells for AB anti-CK7, and absence of staining for AB anti-CK20, anti-GATA3, and anti-mammaglobin, which are indicative of gastric cancer (Figure 6).

The tumor markers were as follows: cancer antigen (CA)-19-9 estimated at 181 U/ml, CA 15-3 at 17 IU/ml and carcinoembryonic antigen (CEA) at 2.6 ng/ml.

The patient received first-line chemotherapy with XELOX regimen (intravenous oxaliplatin 130 mg/m² on day one followed by oral capecitabine 1000 mg/m² twice daily from day one through day 15) every three weeks for three cycles, during treatment, the patient received one blood transfusion for grade 3 anemia. Additionally, the patient experienced two episodes of grade 2 nausea and vomiting with anorexia. There were no residual symptoms of hand-foot syndrome or peripheral neurotoxicity.

The breast metastases responded well to chemotherapy and were reduced in size on subsequent monitoring, but metastatic disease continued to progress in the bone, lymph nodes and peritoneum. The patient underwent second-line chemotherapy with paclitaxel and ramucirumab, and has just received her first cycle.

## Discussion

According to the World Health Organization (WHO), breast cancer is the most common cancer in the world with more than 2.3 million new cases of cancer occurring each year [8].

In Morocco, the incidence is of the order of 51.2 new cases per 100,000 inhabitants per year, with a mortality rate of 4,000 cases, based on the Grand Casablanca Cancer Registry (RCGC) [9]. However, the occurrence of breast metastasis from a second primary site is unusual and remains rare, contralateral breast carcinoma, melanoma, lymphoma, lung or kidney tumor are the most common causes, with gastrointestinal origin being the least common [1,7,10,11].

Gastric tumors rarely metastasize to the breast, however the main sites of metastasis are the peritoneum, liver, lung, lymph nodes and bone [5,7,12], while the major sites of metastasis in the gastric signet ring cell carcinoma subtype, which is the case we are reporting here, are the lymph nodes and peritoneum, the breast is an uncommon site of metastasis and is rarely reported, except in a few clinical cases [4].

In the literature, breast metastases are usually detected after the primary gastric signet cell carcinoma has been diagnosed, but in some cases they can be indicative of the primary [13]. Breast metastases from gastric cancer are more common in women (95%) than in men (5%) [3]. This difference in incidence may be linked to the variation in size, vascularization, hormonal factors, and receptors between the two sexes, as suggested by Georgiannos et al. [14]. It is commonly reported in young women aged between 21 and 70, with a median age of around 46 years, most often in premenopausal women [5,7,13]; in keeping with the literature, our presentation is about a young woman who is 22 years old.

It is still unclear how a tumor in the stomach can spread to the breast. Two different routes of spread have been proposed: lymphatic spread and haematogenous spread [2,5,10,15,16].

The average time between diagnosis of gastric cancer and the development of metastasis to the breast varies from zero to six years, about 40% of breast metastases were detected during or within a year of the primary tumors [5].

The most common signs of breast metastasis are the appearance of a palpable nodule in the breast, which can be single or multiple, with axillary lymph node enlargement occurring in 15%, inflammatory changes or oedema may also be revealing in certain situations [5,7,10,17]. It often occurs on the left side of the breast, specifically at the level of the upper outer quadrant [10]. In 25% of cases, breast involvement is bilateral [18]. So, It's important to consider that any breast changes could indicate the presence of an extra-mammary malignant tumor.

In terms of radiology, the results are not specific enough to distinguish between a breast metastasis and a primary lesion, which makes the diagnosis quite difficult. The radiological images described are heterogeneous. These breast metastases are often located in the upper outer quadrant of the left breast, with bilateral involvement in 25% of cases [17,19], and present as follows; mammography shows an increase in the density of the mammary parenchyma or a thickening of the skin, while the ultrasound shows a round, hypoechoic, well-circumscribed mass with skin or parenchymal involvement, without speculation or microcalcifications, except in the case of ovarian metastatic carcinoma [20], mimicking then a fibroadenoma or benign breast disease [5,17,19-22]. Kwak et al. suggest that the absence of microcalcifications and tumor mass on mammography and ultrasound should be suspicious for breast metastases of signet ring cell carcinoma [1].

Histopathological examination plays a crucial role in differentiating between breast metastases and primary breast cancer. The final diagnosis is established based on immunohistochemical characteristics as a reference standard, this histological character is specific and includes the presence of lymphatic tumor emboli, a peri-lobular or peri-ductal organization without signs of hyperplasia or atypia [17,23,24], absence of elastosis, in situ or intraductal carcinoma, a clear transition at the edge of the tumor, as well as the presence of tumors located in the subcutaneous tissue support the hypothesis of an extra-mammary origin [14,24,25]. Immunohistochemical analysis of breast metastasis from primary gastric cancer usually shows positive staining for CK20, CK7, CEA, and negative staining for GCDFP-15, GATA3, estrogen receptor (ER) and progesterone receptor (PR) [2,7,13,26]. This was the case in our patient.

Therefore, for a more accurate diagnosis, the combination of markers is an ideal choice, especially CDX2, CK20, CK7, RE and RP, as reported by Tot in his analysis [27], allowing to distinguish between primary and secondary breast tumors arising from gastric cancer.

The literature has documented several prognostic factors for gastric cancer with breast metastasis, including tumor size, lymph node involvement, peritoneal metastasis, distant metastasis, and age under 45 years [28].

Tumor histology is also associated with poor prognosis, patients with signet ring cell carcinoma (SIG) were found to have better survival rates than other histologic types followed by adenocarcinoma (AC), poorly differentiated adenocarcinoma (PDA), and at least mucinous adenocarcinoma (MAC), as reported by Ma et al. [7].

To ensure the most appropriate treatment, it is important to distinguish between primary and metastatic breast cancer.

Patients with locally advanced, inoperable or metastatic disease are candidates for palliative cancer treatment; authors state that using specific cancer treatments, such as combinations of chemotherapy drugs, trastuzumab and immunotherapy, improves prognosis and quality of life [5-7,29,30].

Following recommendations from the National Comprehensive Cancer Network (NCCN) and the European Society for Medical Oncology (ESMO) in metastatic gastric cancer, the standard first-line ChT is a platinum fluoropyrimidine doublet with oxaliplatin or cisplatin. For patients with HER2-positive tumours, the first-line treatment consisted of a combination of chemotherapy (FOLFOX and/or XELOX) with trastuzumab. For HER2-negative patients expressing programmed death-ligand 1 (PD-L1), the standard of care is to combine chemotherapy with immunotherapy by adding nivolumab or pembrolizumab to ChT. For second-line or subsequent therapy, depending on the patient's prior therapy and PS, we can use different regimens: ramucirumab with paclitaxel, docetaxel, paclitaxel or irinotecan [31,32].

Breast metastases from signet ring cell gastric carcinoma have a poor prognosis reflected in a lower survival rate, according to the reported cases [28], the survival rate was below one year, with the median survival time being approximately 8.6 months and a high mortality rate of over 80% during the first year [7,33]. He and colleagues reported a median overall survival time ranging from 12 days to 18 months [2]. At this time, the patient is still alive four months after having been diagnosed and started to be treated.

## Conclusions

Gastric tumors are unlikely to cause breast metastases. Diagnosis is mainly based on immunohistochemistry (IHC) and radiology. The use of multiple chemotherapeutic agents and targeted therapy can help in the management of this disease to ensure appropriate treatment, avoid unnecessary surgery and improve quality of life. However, it is associated with a poor prognosis and a shorter life expectancy, which makes breast metastases of gastric cancer a challenging clinical problem.
